# Gypsum-Based Material for Dental Pulp Capping: Effect of Chitosan and BMP-2 on Physical, Mechanical, and Cellular Properties

**DOI:** 10.1155/2018/3804293

**Published:** 2018-07-26

**Authors:** Hasan Subhi, Fazal Reza, Adam Husein, Saaid Ayesh Al Shehadat, Asma-Abdullah Nurul

**Affiliations:** ^1^Conservative Dentistry Unit, School of Dental Sciences, Universiti Sains Malaysia, 16150 Kubang Kerian, Kelantan, Malaysia; ^2^Biomaterials and Prosthodontics Unit, School of Dental Sciences, Universiti Sains Malaysia, 16150 Kubang Kerian, Kelantan, Malaysia; ^3^Department of Prevention and Restorative Dentistry, College of Dental Medicine, University of Sharjah, Sharjah, UAE; ^4^School of Health Sciences, Universiti Sains Malaysia, 16150 Kubang Kerian, Kelantan, Malaysia

## Abstract

Effective pulp capping material must be biocompatible and have the ability to induce dentin bridge formation as well as having suitable physical and mechanical properties; however, many current materials do not satisfy the clinical requirements. This study aimed to assess the physical and mechanical properties of gypsum-based chitosan material (Gp-CT) and to evaluate its effects on cellular properties of stem cells from human exfoliated deciduous teeth (SHED). The experimental material was prepared with different concentrations of chitosan (CT) with or without BMP-2. Then, setting time, compressive strength, and pH were determined. In addition, cell viability, alkaline phosphatase (ALP) activity, and cell attachment were assessed. The setting time, compressive strength, and pH obtained were 4.1–6.6 min, 2.63–5.83 MPa, and 6.5–5.7, respectively. The cell viability to gypsum (Gp) with different CT concentrations was similar to that of the control on day 1 but statistically different from that of Gp alone on day 3. The ALP activity of SHED was significantly higher (p < 0.05) in CT- and BMP-2-containing materials than those in the control and Dycal at days 3 and 14. The scanning electron microscopy (SEM) image revealed that flattened cells were distributed across and adhered to the material surface. In conclusion, Gp-CT material shows promise as a potential material for direct pulp capping.

## 1. Introduction

Direct pulp capping is a process of covering the exposed vital pulp with a material to maintain its vitality and preserve its biological and functional activities. The capping material is applied to the exposed site to induce the formation of reparative dentin. Effective pulp capping materials must be biocompatible and have the ability to induce dentin bridge formation; however, many current materials do not satisfy the clinical requirements.

A number of pulp capping materials have been introduced. Calcium hydroxide was the material of choice for pulp capping because of its ability to stimulate hard-tissue deposition and high antibacterial efficiency [1]. However, this compound exhibits several limitations, such as poor sealing, formation of “tunnel defects” in dentin barrier, and gradual degradation [2–4]. Calcium silicate-based materials, such as mineral trioxide aggregate (MTA), and Biodentine have been used in pulp capping. MTA is a suitable material for pulp capping, demonstrating a higher success rate than calcium hydroxide [5]. MTA is superior to conventional pulp capping materials but exhibits poor handling properties, high cost, and slow setting time [6–8].

Studies on material development have progressed. Bio-inspired materials are being developed to preserve and induce the regeneration at the application site. Calcium sulfate or “gypsum” is widely used in dentistry and orthopedics because of its biocompatibility [9]. Gp can be utilized as a resorbable scaffold to support bone growth and release drugs associated with graft materials [10]. Numerous studies have investigated the effects of CT-incorporated Gp compound in tissue regeneration; this compound exhibits biocompatibility and stimulates osteogenesis/odontogenesis and is thus studied for bone formation in orthopedics [11–13] and as a pulp capping material in dentistry [14, 15]. It promotes osteoinduction and bone formation when incorporated with bone morphogenetic protein-2 (BMP-2) [16, 17]. Therefore, in the present study we evaluated the potential of Gp-CT material with BMP-2 for direct pulp capping.

This study aimed to determine the setting time, compressive strength, and pH of an experimental Gp-CT and to evaluate its effects on cell viability, ALP activity, and cell attachment when incorporated with BMP-2 and compare the material with Dycal.

## 2. Materials and Methods

### 2.1. Preparation of the Materials

PROTASAN UP CL 113 is a well-characterized water-soluble chitosan chloride referred to as CT in this study (NovaMatrix, Norway; 75%–90% degree of deacetylation; molecular weight of 50,000–150,000 g/mol). Calcium sulfate dihydrate (Sigma-Aldrich, India) was heated at 110°C for 3 h in an electric oven (Universal Oven Memmert Life 600, Schwabach, Germany) for conversion into a hemihydrate form (CaSO_4_*∙*1/2 H_2_O) [14]. BMP-2 was subjected to ALP assay and SEM to evaluate its effects on SHED.

BMP-2 (AllCells, USA) was impregnated in distilled water to obtain a concentration of 50 ng/ml and form BMP-2 solution. CT powder was then added and dissolved to prepare five different concentrations of CT solution of 10%, 5%, 2.5%, 1%, and 0% w/v. Gp was mixed with the CT solutions to prepare the material. The ratio of CT solution to Gp is 0.6 ml/g. Dycal (DENTSPLY Caulk, USA) was prepared in accordance with the manufacturers' instructions.

### 2.2. Setting Time

Molds with a diameter of 10 mm and a height of 5 mm were filled with freshly mixed experimental materials and Dycal. The samples were then stored at 37 ± 1°C and at relative humidity of at least 95%. A Vicat needle with 300 g in weight and a needle having a flat ended cylindrical tip of 1.0 ± 0.02 mm in diameter was applied carefully to the surface of the sample and indentations were made at 15 sec intervals in new locations. The setting time was recorded from the start of mixing to the nearest 15 sec interval at which the needle failed to penetrate the sample completely. Six samples of each tested material were evaluated. The average of the individual trials was examined.

### 2.3. Compressive Strength

The materials and Dycal were mixed, placed in molds with a diameter of 6 mm and a height of 12 mm, and stored at 37°C for 24 h. The molds were then removed, and the specimens were placed between the platens of a universal testing machine (Instron 8874, USA). The load was applied along the long axis of the specimens at a crosshead speed of 1 mm/min. The compressive strength was recorded at the point of the specimen fracture. Six samples of each material were evaluated. The compressive strength was calculated in megapascals according to

(1) $$\begin{array}{c} C=\frac{4P}{\pi D^{2}} \end{array}$$

  P is the maximum load applied in Newton.
  D is the mean diameter of the specimen in millimeters.

### 2.4. pH Value

The materials and Dycal were mixed and placed into plastic wells at approximately 10 mm in diameter and 5 mm deep. A digital pH meter (FieldScout SoilStik, Spectrum Technologies, Inc., China) was used to measure the pH levels. A small amount of distilled water was dispensed to wet the surface of the materials before the electrode was placed onto the surface of the set material.

The pH measurements of the materials were recorded at 10 min, 2 h, and 24 h. Prior to measurements, the device was calibrated using buffer solutions with pH 4 and 7 throughout the experiment. Six measurements for each material were recorded for analysis.

### 2.5. Culture of Stem Cells from Human Exfoliated Deciduous Teeth

SHED were purchased from AllCells, USA. The cells at passage 8 were cultured in Alpha minimum essential medium [*α*-MEM; Invitrogen, USA (GIBCO)] supplemented with 10% fetal bovine serum [FBS; Invitrogen, USA (GIBCO)] and 1% penicillin-streptomycin solution [Invitrogen, USA (GIBCO)]. The cells were incubated in a humidified incubator with CO_2_ at 37°C.

### 2.6. MTS Assay

SHED were seeded in 24-well plates (Nunc™, Denmark) at 1 × 10^4^ cells/800 *μ*l of growth medium in each well and incubated for 24 h at 37°C in 5% CO_2_. The freshly mixed materials and Dycal were placed in acrylic molds with a diameter of 5 mm and a height of 3 mm to fabricate the material discs. After the materials were set, the material discs were released and sterilized with UV light in a biosafety cabinet (Labconco, USA) for 30 min (15 min for each side).

Afterward, the material discs were placed in the wells under aseptic conditions as described previously [18]. SHED alone served as a control group. Cell viability was evaluated through a 3-(4,5-dimethyl-thiazol-2-yl)-5-(3-carboxy-methoxy-phenyl)-2-(4-sulpho-phenyl)-2H tetrazolium assay. On days 1 and 3, the material discs and the culture medium were removed. Afterward, 300 *μ*l of culture medium and 60 *μ*l of MTS (Promega, USA) were added to each well and incubated for 2 h. Then, 100 *μ*l of MTS solution from each well was transferred into 96-well plate in triplicate for each material. Absorbance was measured using ELISA reader (Tecan, Japan) at a wavelength of 490 nm. The cell viability was calculated according to

(2) $$\begin{array}{c} Cell\;\;viability\;\left(\;\%\;\right)=\frac{\text{absorbance of samples}}{\text{absorbance of control}}\times 100 \end{array}$$

### 2.7. Alkaline Phosphatase Activity

ALP activity was evaluated using a colorimetric ALP assay (Randox, UK). In this procedure, the substrate* p*-nitrophenyl phosphate is hydrolyzed by alkaline phosphatase from the cells in the presence of magnesium ions to form* p*-nitrophenol and finally measured at 405 nm.

SHED at a concentration of (2.5 × 10^4^ cells/1 mL of medium per well) were cultured in a 12-well plate and incubated at 37°C in 5% CO_2_ for 24 h. The material disc as prepared in MTS assay of two groups with or without BMP-2 incorporation was placed in each well. SHED alone served as a control group, and the culture medium was changed every 3 days. At 3 and 14 days of incubation, the material discs were removed, and 100 *μ*l of the supernatant was transferred into a new 96-well plate in quadruplicate for each material. Afterward, 15 *μ*l of the ALP substrate pNPP was added to each well and incubated for 1 h in an incubator with 5% CO_2_. Then, 20 *μ*l of 1 M (NaOH) stop solution was added, and the absorbance was measured using ELISA reader at 405 nm.

### 2.8. Scanning Electron Microscopy

The material discs with a diameter of 12 mm and a height of 2 mm were placed in a 24-well plate. SHED were then seeded at 5 × 10^4^ cells on the materials discs and immersed in the medium. The plates were incubated for 3 days at 37°C in 5% CO_2_. The material discs were washed with distilled water and fixed with 2.5% glutaraldehyde for 2 h at 4°C. Then, the samples were dehydrated in ethanol at the concentrations of 30%, 50%, 70%, 80%, 90%, and 100% for 10 min at each concentration and later dried in a desiccator (FSD-380, TECH-LAB SCIENTIFIC, Malaysia). Finally, the samples were sputter coated with gold and examined under SEM (Phenom-World, Netherland).

### 2.9. Statistical Analysis

Statistical analyses were performed using SPSS (Version 22.0; SPSS, Chicago, IL). Statistical significance was evaluated by one-way analysis of variance (ANOVA) followed by Dunnett T3 post hoc test for multiple comparisons in setting time, compressive strength, MTS assay, and ALP activity. Statistical analysis of the pH values was performed by repeated measures analysis of variance (ANOVA). A value of p < 0.05 was considered statistically significant.

## 3. Results

### 3.1. Setting Time

**Figure 1:**
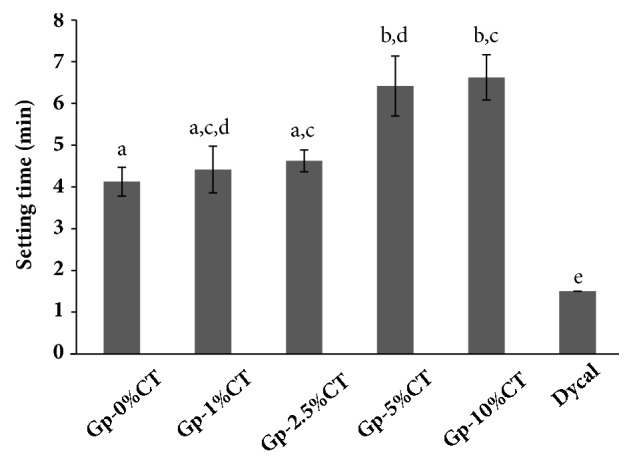
The setting time of Gp-CT materials and Dycal. The error bars represent the standard deviation. Bars with different letters are statistically significant at p < 0.05.

The setting time of the materials and Dycal is listed in Figure 1. The setting time of the experimental materials ranged from 4.1 min to 6.6 min; the setting time was longer in the higher CT concentration. This result indicated that setting time was altered in a dose-dependent manner. Our findings further showed that setting time of Gp-5%CT and Gp-10%CT was significantly longer than Gp-0%CT (p < 0.05). Dycal showed a setting time of 1.5 min, and this result significantly differed from the setting time of all the tested materials (p < 0.05).

### 3.2. Compressive Strength

**Figure 2:**
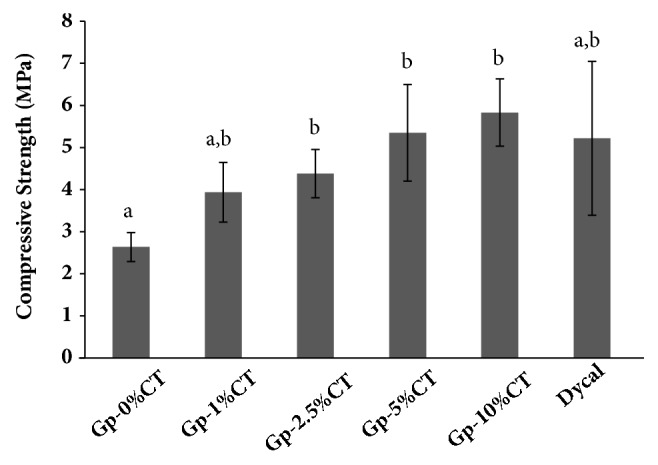
Compressive strength of the Gp-CT materials and Dycal. The error bars represent the standard deviation. Bars with different letters are statistically significant at p < 0.05.

The compressive strength of the materials and Dycal is listed in Figure 2. The compressive strength of the experimental materials ranged from 2.63 MPa to 5.83 MPa. Results indicated that a high compressive strength corresponded to higher CT concentration. Compressive strength of Gp-2.5%CT, Gp-5%CT, and Gp-10%CT was significantly higher than Gp-0%CT. Interestingly, compressive strength of Gp-5%CT and Gp-10%CT was comparable with that of Dycal.

### 3.3. PH Value

**Figure 3:**
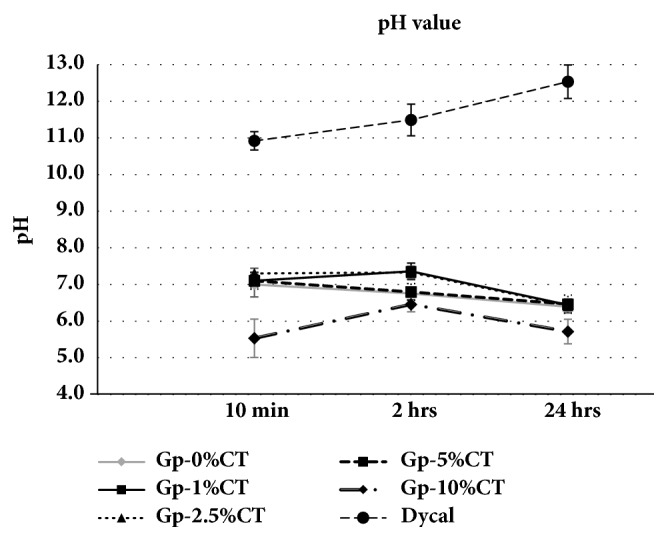
pH values of Gp-CT materials and Dycal at 10 min, 2 hrs, and 24 hrs (mean ± SD).

The pH of the materials and Dycal is listed in Figure 3. The pH of the Gp-CT materials at 24 h ranged from 6.5 to 5.7, with a tendency toward lower pH with higher CT concentration. Similar tendency was observed at 10 min with slightly higher pH than that of 24 h while consistently high alkaline pH of approximately 12.5 was observed with Dycal at 24 h with significant differences with the measurement at 10 min.

### 3.4. MTS Assay

**Figure 4:**
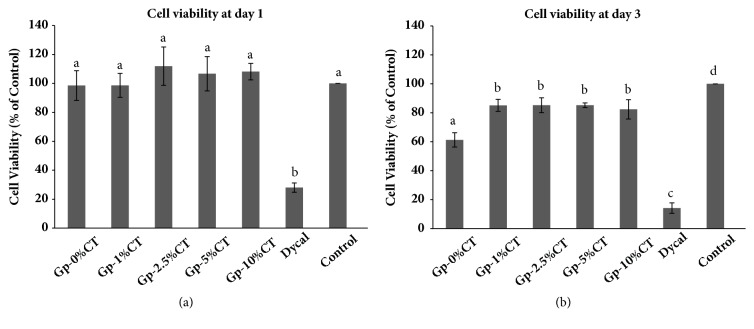
Cell viability values of SHED cultured with various concentrations of Gp-CT and Dycal after (a) 1 day and (b) 3 days as measured by MTS assay. The error bars represent the standard deviation. Bars with different letters are statistically significant at p < 0.05.

Figures 4(a) and 4(b) show the viability of the SHED treated with the experimental material and Dycal. On day 1, the cell viability of the experimental materials ranged from 98.5% to 111.9%. There was no significant difference of the viability from the cells cultured on experimental materials compared to the control group at day 1. However, cell viability of the experimental materials showed decreasing pattern at day 3. Interestingly, SHED cultured with Gp-CT showed higher viability compared to Gp alone. In contrast, Dycal demonstrated cytotoxic effects to the SHED with the cell viabilities of 28% and 14.2% on days 1 and 3, respectively, and the viability was significantly lower than all the materials and the control (p < 0.05).

### 3.5. Alkaline Phosphatase Activity

**Figure 5:**
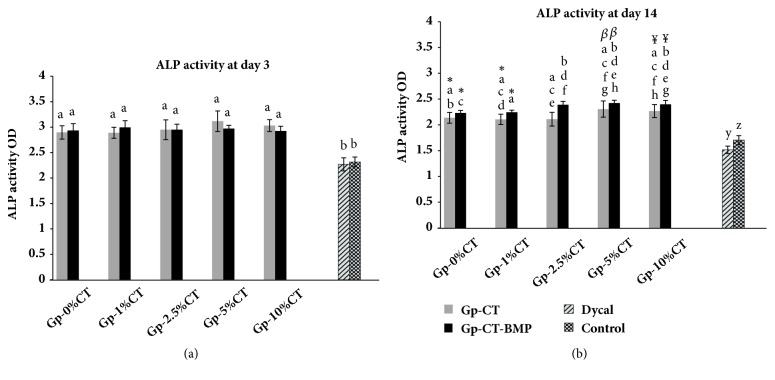
ALP activity of the Gp-CT and Dycal on SHED after (a) 3 days and (b) 14 days of incubation. The Gp-CT was cultured with or without BMP-2. The error bars represent the standard deviation. Bars with different letters are statistically significant at p < 0.05.

The ALP activity of the SHED cultured with the material and Dycal is shown in Figures 5(a) and 5(b). On day 3, SHED cultured with the experimental materials showed statistically higher ALP activity than the control groups and Dycal (p < 0.001). No significant differences could be noted in the ALP activities of Gp-CT (at various concentrations) prepared in the presence of BMP-2 when compared with Gp-CT without BMP-2.

On day 14, SHED seeded on various concentrations of Gp-CT with or without BMP-2 demonstrated significantly higher ALP activity than the control group and Dycal. SHED seeded on Gp-CT-BMP with various CT concentrations showed higher ALP activity than those on Gp-0%CT-BMP. In addition, Gp-1%CT-BMP exhibited significantly lower ALP activity than Gp-2.5%CT-BMP, Gp-5%CT-BMP, and Gp-10%CT-BMP. Interestingly, Gp-2.5%CT-BMP demonstrated significantly higher ALP activity than Gp-2.5%CT alone (p* = *0.016).

### 3.6. Scanning Electron Microscopic Analysis

**Figure 6:**
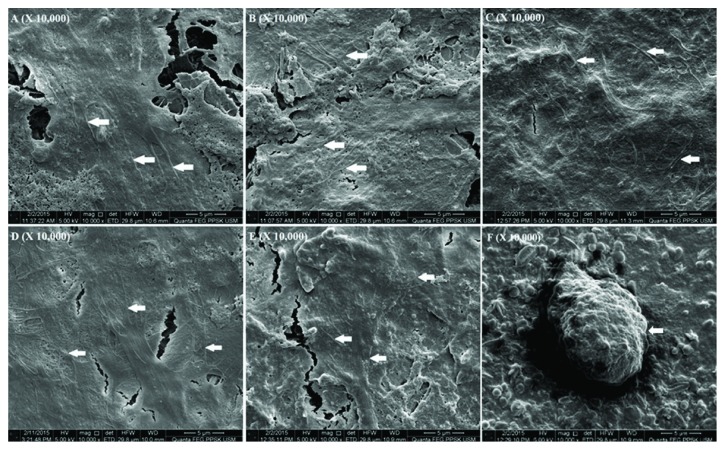
SEM analysis of SHED attachment and growth following 3 days of incubation on (A) Gp-0%CT-BMP material, (B) Gp-1%CT-BMP material, (C) Gp-2.5%CT-BMP material, (D) Gp-5%CT-BMP material, and (E) Gp-10%CT-BMP material. Pictures showing numerous thin cytoplasmic extensions of the cells (white arrows); (F) SEM analysis showing rounded nonliving cell on Dycal (white arrows).

Results revealed that flattened SHED spread across the surface and adhered to the underlying material through their cytoplasmic processes. Multiple thin cytoplasmic extensions were extended from the cells and projected to the surrounding adjacent cell surface (Figures 6(A)–6(E)). By contrast, few rounded cells were observed on Dycal (Figure 6(F)).

## 4. Discussion

Direct pulp capping is important to avoid more invasive and resource-intensive procedures of conventional root canal treatment. It initiates the formation of dentin by reparative dentinogenesis through a series of processes that begin with the differentiation of dental pulp stem cells into odontoblast-like cells. Growth factors, such as BMP-2, play an important role in dentin regeneration and dentinogenesis [19]. The ability of pulp capping materials to induce SHED differentiation is critical and is mainly determined by interactions between the materials and cells. SHED provide an eligible cell source for regenerative endodontics because these cells originate from dental tissues and differentiate successfully into odontoblast-like cells [20].

Results showed that the setting time increased with increasing CT concentration, indicating dose-dependent changes. That may be related to the high viscosity of the CT-containing solution of materials, resulting in obstruction of ion diffusion in the matrix [21]. The setting time of Gp-CT meets the guideline of ISO 3107 guideline for zinc oxide/eugenol cements and zinc oxide/noneugenol which recommends a setting time range of 4–10 min [22].

The mechanical properties of Gp can be affected by the compression in microstructure and size (thickness and length) of the entangled crystals [23]. The compressive strength increased with increasing CT concentration. A high CT concentration yields a highly viscous CT solution, which reduces the porosity of hardened materials [24] and also influences Gp crystallization by producing thick and protracted crystals of tightly locked Gp-CT materials [14]. Similarly, Ślósarczyk et al. [24] have stated that the compressive strength of the cement is influenced by the concentration of CT solution added.

The pH of Gp-CT after 24 h ranged from 6.5 to 5.7, and a lower pH was obtained when using higher CT concentration (Gp-10%CT). The pH values recorded are considered suitable for pulp capping because they are ideal for cell culture [25] and may form a necrotic zone-free area on the application site.

The cell viability was enhanced when CT added to the material. Compared to the control, Gp-CT demonstrated no cytotoxic effects, and an enhanced cell viability to Gp-2.5%CT, Gp-5%CT, and Gp-10%CT was observed on day 1, while a significantly declined cell viability was reported on day 3. The percentage of cell viability in treatment with Gp-0%CT decreased to 61.3% on day 3, indicating the lowest cell viability. Gp is an osteoinductive and passive-osteoconductive material because of its unique crystal structure and high calcium content [26]. Takita et al. [25] have stated that calcium-containing elution increases the proliferation of human dental pulp cells in dose-dependent manner. By contrast, Dycal induced high cytotoxicity to the cells, similar to the previous findings [27, 28]; the high cytotoxicity of Dycal is related to the high pH.

SHED cultured with various concentrations of Gp-CT with or without BMP-2 demonstrated higher ALP activity than the control and Dycal on days 3 and 14. Gp-2.5%CT incorporated with BMP-2 yielded a higher ALP activity on day 14 when compared to Gp-2.5%CT alone. The experimental material enhanced the concentration of ALP released outside the cells; this finding may be attributed to the influence of the experimental material on the osteogenic differentiation of the cells. Similarly, Lisa et al. [29] evaluated the potential of CT to induce osteogenic differentiation of macaque dental pulp stem cells and revealed that CT possibly promoted the release of ALP into the medium. Calcium sulfate provides a source of calcium ions, which accelerates the mineralization and regeneration of the tissue [30]. In addition, the enhancement of ALP activity by CT was reported in many studies [29, 31].

The cells were apparently healthy under SEM after 3 days of seeding on the material surface. The cells spread and adhered to the materials at all CT concentrations. Cell adhesion is implicated in cell growth, differentiation, and proliferation; this complicated process is also involved in wound healing [32]. The cells appeared flat and exhibited well-defined cytoplasmic extensions. The presence of cytoplasmic extensions is necessary to form three-dimensional networks inside the materials. Lazáry et al. [26] have found that osteoblasts adhered to the surface of Gp because of its physical structure. Moreover, Gp contains a number of small crystals located side by side; these crystals form a large molecular surface. By contrast, few rounded and detached cells were distributed on the surface of Dycal. These cells are dead and comparable with those described in a previous study [33].

## 5. Conclusions

The results of our study showed that Gp-CT material demonstrated acceptable setting time and compressive strength for clinical applications and also stable pH values. In addition, the material demonstrated a potential to promote the proliferation, differentiation, and attachment of SHED* in vitro*. However, the mechanism through which Gp-CT stimulates the osteogenic/odontogenic differentiation of SHED should be further investigated.

## Data Availability

The mean ± SD data in excel file used to support the findings of this study are available from the corresponding author upon request.
